# Outcomes with non-small cell lung cancer and brain-only metastasis

**DOI:** 10.1016/j.heliyon.2024.e37082

**Published:** 2024-08-29

**Authors:** Sabine Schmid, Miguel Garcia, Luna Zhan, Sierra Cheng, Khaleeq Khan, Maisha Chowdhury, Amir Sabouhanian, Joshua Herman, Preet Walia, Evan Strom, M. Catherine Brown, Devalben Patel, Wei Xu, Frances A. Shepherd, Adrian G. Sacher, Natasha B. Leighl, Penelope A. Bradbury, Geoffrey Liu, David Shultz

**Affiliations:** a Department of Medical Oncology, Inselspital, Bern University Hospital, University of Bern, Switzerland; b University Health Network, Princess Margaret Cancer Centre, Toronto, Canada; c Ramon y Cajal University Hospital, Madrid, Spain

**Keywords:** NSCLC, Brain-only disease, Metastatic failure patterns, Prognosis, Local treatment of the primary

## Abstract

**Background:**

We evaluated outcomes in non-small cell lung cancer (NSCLC) patients who presented with brain-only metastatic (BOM) disease overall and by EGFR/ALK mutation status.

**Methods:**

We analyzed clinico-demographic, treatment and survival data for all NSCLC patients who presented to our center between 2014 and 2016 with BOM as their first presentation of metastatic disease. Differences in overall survival (OS) were evaluated using log-rank tests for NSCLC wildtype (NSCLCwt*) versus* NSCLC with an ALK-rearrangement/EGFR-mutation (NSCLCmut+).

**Results:**

Of 109 patients with BOM, median age was 68 years; 51 % were female; 69 % Caucasian; 76 % ever-smoker; 76 % adenocarcinoma; and 25 % NSCLCmut+. While 41 patients (38 %) had subsequent brain-only progressive disease (PD), 22 (20 %) developed extracranial metastases. A higher proportion of NSCLCmut+ (*vs* –wt) subsequently progressed outside the brain (37 % vs 15 %, p = 0.03). Median time-to-first-extracranial-metastases was 8.5 (NSCLCmut+*) vs* 21.0 months (NSCLCwt; p = 0.23).

With 17.7 months median follow-up, median-OS was 15.9 months [95%CI: 11.5–21.3; all patients]; 12.3 [7.4–18.4; NSCLCwt] and 38.9 [21.3-not reached (NR); NSCLCmut+] (p = 0.09). In 33 of 80 patients with *de novo* BOM, the primary tumor was treated with surgery or radiotherapy. In patients with NSCLCwt, there was no OS benefit associated with local lung tumor treatment (p = 0.68), whereas in NSCLCmut + pts, local lung tumor treatment correlated with greater OS (median-OS NR vs 21.5 months; p = 0.05).

**Conclusion:**

In patients with NSCLCwt with BOM, we observed a -predominant pattern of brain-only secondary progression, however patients with NSCLCmut + more often progressed extracranially. In patients with NSCLCmut+ and BOM, definitive primary tumor treatment correlated with improved survival.

## Highlights

• We observed favourable outcomes in patients with NSCLC and brain only metastatic disease.
• Significant differences in metastatic failure patterns according to mutational status were seen.
• Primary tumor treatment was associated with improved survival in patients with NSCLCmut+.

## 1 Background

The brain is a common site of metastasis in non-small-cell lung cancer (NSCLC); approximately 20 % of patients have brain metastases (BrM) at diagnosis [1] and 40 % develop BrM during their disease course [2].

BrM from NSCLC were previously associated with a median overall survival (OS) of approximately 7 months before the advent of targeted treatments, stereotactic radiosurgery (SRS), and improvements in surgical and supportive care [3]. In an older, multi-institutional retrospective database including 1833 patients with BrM from NSCLC, the first Graded Prognostic Assessment (GPA) tool included age, performance status, presence of extracranial metastases and number of brain metastases. However, with more widespread use of molecular testing and targeted treatments [4], a lung-specific GPA in 2017 added the presence of *EGFR* mutations or *ALK* rearrangements as additional prognostic factors [5]. The lung-specific GPA was recently updated to include PD-L1 expression as an additional prognostic factor [6]. In these models, the presence of extracranial metastasis remains a poor prognostic factor for patients with BrM; however, it is unknown whether prognosis for patients with brain-only metastasis (BOM) is worse than for patients with extracranial disease alone.

With regard to BrM treatments, for decades, whole-brain radiation treatment (WBRT) represented the standard of care; however, definitive and adjuvant SRS have become increasingly common in recent years. Current ASCO-SNO-ASTRO BrM guidelines [7] recommends radiotherapy for the majority of patients, except for patients with asymptomatic lesions and a Karnofsky Performance Score (KPS) of ≤50 % or in patients with a KPS <70 and no systemic treatment options. Generally, SRS alone is recommended for patients with 1–4 metastases, whereas in patients with >4 metastases SRS with WBRT, or SRS or WBRT alone are both considered reasonable options. In patients with a superior prognosis or CNS active systemic options, SRS is preferred. These recommendations continue to evolve. Surgery is mainly recommended when BrM cause symptoms from mass effect or when histology is needed. Finally, local treatment may be deferred in patients who are treated with systemic therapies with known high CNS activity. However, most studies that underlie these guidelines did not specifically include patients with BOM and therefore extrapolation of these guidelines to this population, especially with regard to systemic therapy, is potentially fraught.

In patients with oligometastatic disease, usually defined as <5 metastatic lesions, an aggressive approach with radical treatment to all sites has been increasingly investigated primarily in mixed tumor-type studies [8]. In a prior retrospective analysis of NSCLC patients presenting with brain oligometastasis at the time of diagnosis, aggressive thoracic treatment plus CNS-directed therapy correlated with an OS-benefit compared to patients who did not receive radical treatment to the primary tumor [9]. A recent meta-analysis of seven trials including NSCLC patients from 3 randomized phase II, 1 phase III, and 3 propensity-matched retrospective studies suggested that local consolidative therapy, especially in patients with oligometastatic disease, associated with improved PFS and OS. That benefit was similar among patients with EGFR-mutation (2 trials) compared to wildtype NSCLC (5 trials). Most of these studies were conducted in the pre-immunotherapy era [10].

NSCLC patients with BOM represent a distinct patient population, possibly with a different underlying tumor biology and prognosis compared to patients with brain and extracranial metastases or extracranial metastases alone. Optimal management with regard to local and systemic treatment is uncertain and it is not known whether BOM confers a worse prognosis compared to extracranial metastases alone. Furthermore, metastatic failure patterns and potential implications for treatment monitoring in this special patient population have not yet been reported.

The aim of this analysis was to characterize potentially distinct clinicopathologic features, current treatment as well as metastatic failure patterns and associated outcomes in this specific patient population with NSCLC and BOM to help guide treatment decisions and treatment monitoring strategies in these patients.

## 2 Methods

The primary objective of the study was to describe real-world treatment patterns and outcomes for patients with BOM from NSCLC at initial or recurrent stage IV disease. A secondary objective was to assess the outcomes for this population compared to alternative metastatic patterns at the time of first metastatic presentation (extracranial metastases only; extracranial and BrM). The University Health Network (UHN) institutional research ethics board approved this study.

### 2.1 Patients

All patients with histologically confirmed advanced-stage NSCLC diagnosed between January 1, 2014 and December 31, 2016 seen at least once at UHN for their NSCLC diagnosis were included. Patients identified with BOM (main study cohort) were divided further into NSCLC wildtype (NSCLCwt) versus NSCLC with an ALK/EGFR alteration (NSCLCmut+) cohort.

Detailed manual data abstraction via retrospective chart review was performed to collect patient clinico-demographic variables including age, sex, ethnicity, AJCC/UICC TNM stage, histology, presence/absence of molecular alterations, smoking history and survival endpoints. Extracted data also included systemic regimen(s), trial participation, dates of disease progression, and metastatic locations at stage IV diagnosis and disease progression.

For the patients with BOM, Eastern Cooperative Oncology Group (ECOG) performance status (PS) at baseline, number of BrM at baseline and detailed radiotherapy information were abstracted. PS was documented as physician-assessed or (if not available) abstractor-assessed based on documentation in clinical notes.

Given this was a retrospective analysis, response assessment was investigator-assessed; no formal tumor assessment per RECIST was performed.

From 2014 to 2016, ALK/EGFR testing was performed routinely for all patients with adenocarcinoma; broader panels were routinely applied after 2016, therefore, information on other molecular alterations such as KRAS status and ROS1 are mostly unavailable.

### 2.2 Statistical analysis

Descriptive summaries of patient clinico-demographic characteristics were reported, stratified by sites of metastasis at diagnosis. Secondarily, within the BOM population, patient clinico-demographic characteristics were stratified by whether there was local treatment (radiation and/or surgical resection) to the primary tumor, and by patterns of metastatic failure during follow-up. Patterns of failure during follow-up were divided into four groups: developed brain progressions only; developed other new extracranial metastasis and brain progression; developed other new extracranial metastasis without any brain progressions; neither brain progression nor other new extracranial metastasis. For patients without either brain progression or new extracranial metastases, we subsequently indicated whether the patient exhibited clinical progression of their existing metastatic disease at any time, up to the last known follow-up. Comparisons between groups were conducted using Pearson's chi-squared tests and Fisher's Exact tests for categorical variables, and Kruskal Wallis H-tests for continuous variables.

Swimmer plots were used to visualize individual patient patterns of failure and treatment received from the date of initial stage IV diagnosis to the date of last follow-up or death.

Overall survival between groups was evaluated using Kaplan-Meier curves and Mantel-Haenszel log-rank tests. Additionally, within the *de novo* brain-only metastatic disease population (i.e. initially diagnosed with both stage IV disease and brain-metastases), univariable and multivariable Cox proportional-hazard models were used to explore the association between whether local treatment to the primary tumor was given and overall survival, controlling for prognostic factors determined a priori; these factors included age, sex, NSCLCwt *versus* NSCLCmut+, systemic treatment within 4 months of diagnosis, and number of brain lesions at stage IV diagnosis (1–2 vs. >2 lesions). The multivariable model was created through Hosmer-Lemeshow's augmented backward selection and purposeful clinical variable selection: all covariates were included and removed stepwise; covariates were not removed if removal resulted in a >10 % change in the effect size of other covariates of interest. Sex and number of brain lesions at stage IV diagnosis subsequently were dropped from the final model. In two additional sensitivity analysis models, we, 1) additionally controlled for T stage and N stage at the time of diagnosis, and, 2) restricted the analyses to patients treated locally for their brain metastases. Collinearity between covariates was examined with a variance inflated factor test, while the proportional hazard assumption was evaluated using Schoenfeld residual plots.

Statistical significance was defined as P < 0.05. All tests were 2-sided. All analyses were conducted using R, version 4.1.0.

## 3 Results

**Study population:** Overall, 947 patients with metastatic NSCLC were included (either at diagnosis or after progression from initial non-stage IV disease), of which 109 (12 %) patients presented with BOM disease, 215 (23 %) presented with brain plus extracranial metastatic sites, and 623 (66 %) presented with extracranial metastatic disease only (Table 1).

**Table 1:** Patient with brain only-metastatic disease at diagnosis of stage IV compared to overall cohort of patients with NSCLC at stage IV depending on initial metastatic pattern.

Covariate	Category	Overall cohort	Brain only	Extracranial only	Brain and Extracranial
Total N (%)		947 (100)	109 (12)	623 (66)	215 (23)
Age at stage IV	Median (IQR)	68.2 (60.7–76.4)	68.1 (59.1–73.7)	69.3 (61.4–77.3)	66.6 (58.4–74.4)
Sex	Female	422 (45)	55 (51)	262 (42)	105 (49)
	Male	525 (56)	54 (50)	361 (58)	110 (51)
Ethnicity	Asian	167 (24)	19 (24)	99 (22)	49(30)
	Caucasian	440 (64)	55 (69)	289 (64)	96 (59)
	Other	86 (12)	6 (8)	61 (14)	19 (12)
	Missing	254	29	174	51
Smoking Status	Ever	668 (73)	79 (76)	449 (74)	140 (68)
	Never	254 (28)	25 (24)	161 (27)	68 (33)
	Missing	25	5	13	7
Histology	Adenocarcinoma wt	484 (51)	56 (51)	314 (50)	114 (53)
	EGFR/ALK pos.	246 (26)	27 (25)	144 (23)	75 (35)
	Squamous Cell	167 (18)	18 (17)	132 (21)	17 (8)
	Large Cell carcinoma	50 (5)	8 (7)	33 (5)	9 (4)
Stage at initial diagnosis	I	54 (6)	3 (3)	48 (7)	3 (1)
	II	51 (5)	9 (8)	39 (6)	3 (1)
	III	103 (11)	17 (16)	76 (12)	10 (5)
	IV	739 (78)	80 (73)	460 (74)	199 (93)

Patients within BOM disease were median 68.1 years of age (range 59.1–73.7); 55 (51 %) female; 55 (69 %) Caucasian, and 79 (76 %) ever-smokers. Most patients had adenocarcinomas (n = 83, 76 %) and 27 (25 %) were found to have an oncogenic driver alteration (EGFR: n = 29, ALK: n = 4); 80 (73 %) presented with BOM disease at initial diagnosis (*de novo*) and 29 (27 %) were diagnosed with BOM as recurrent disease following initial diagnosis with non-Stage IV disease (Table 1).

When compared to the overall population of metastatic NSCLC patients, no relevant differences in baseline characteristics were identified comparing BOM to non-BOM patients (Table 1).

### 3.1 Initial therapeutic management of BOM disease (Fig. 1)

The majority of patients (n = 93, 85 %) received upfront local BrM treatment: 64 (59 %) were treated with SRS ± surgery and 29 (27 %) received WBRT ( ± surgery). Overall, 25 (23 %) underwent surgery, 12 (11 %) underwent surgery only, 13 (12 %) underwent surgery plus radiotherapy; and 16 (15 %) patients received no upfront local BrM treatment.

The majority of patients (n = 48, 75 %) treated with SRS or surgery had 1-2 BrM lesions at diagnosis while 1 presented with >10 lesions (2 %). Patients treated with upfront WBRT were more likely to have greater than 10 lesions (n = 14, 50 %).

Forty-two patients (39 %) received systemic therapy within 4 months of BrM diagnosis, whereas 60 (55 %) did not (for 7 patients systemic treatment information was unavailable). Initial systemic treatment (within 4 months of PrM diagnosis) consisted of chemotherapy in 26 (62 %) patients, immunotherapy in two (5 %) and targeted therapy in 12 (29 %).

Patients with NSCLCwt (all histologies), compared to patients with NSCLCmut+, were less likely to receive upfront systemic therapy (29 % vs. 67 %, p < 0.01), whereas the overall rate of initial local brain treatment was comparable (88 % vs 78 %, P = 0.22): 65 % vs 41 % (P = 0.04) were managed with SRS or surgery, 23 % vs 37 % (p = 0.21) received WBRT (Supplementary Fig. 1).

**Fig. 1:**
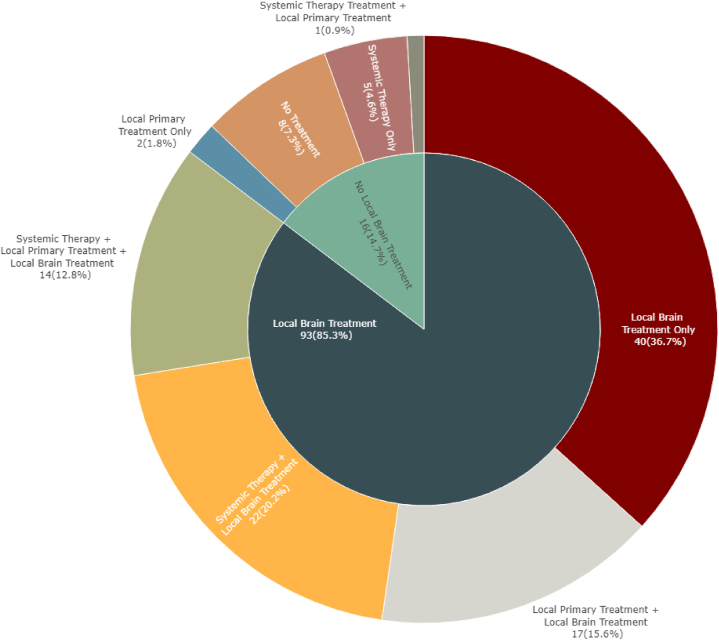
Treatment patterns of 109 patients with BOM at initial diagnosis (within 4 months of stage IV diagnosis). - Within 101 patients receiving at least 1 treatment. −8 Patients received no treatment (3 Surveillance, 3 Refused, 1 Deceased, 1 Lost to follow-up).

Among 80 patients presenting with *de novo* BOM, 33 (41 %) underwent local treatment directed at their primary tumor. Overall baseline characteristics of these patients were not significantly different from patients who did not receive local thoracic treatment, including intrathoracic tumor volume as represented by T and N-Stage (missing in 7 and 5 patients, respectively). Of note, 24 (73 %) patients who had primary local tumor treatment presented with only 1–2 BrM; in contrast, only 22 (47 %) of patients without local treatment to the primary tumor and 1–2 BrM (Supplementary Table 1). In the NSCLCwt group, 28 patients (46 %) received treatment to the primary tumor compared to 5 patients with NSCLCMut+ (26 %). Local treatment to the primary tumor consisted of surgery in 9 (27 %) patients, concurrent chemoradiation in 6 (18 %) and radiation alone in 18 (55 %) patients (n = 4 full dose RT; n = 14 palliative dose RT) (see Fig. 1).

### 3.2 Patterns of failure and subsequent therapies in BOM patients

**Fig. 2:**
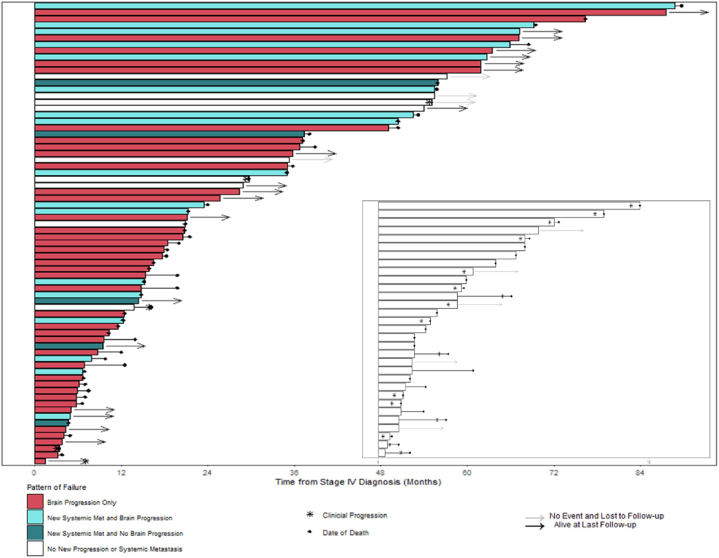
Swimmers plot of all patients illustrating metastatic failure patterns of patients with initially brain-only disease. - Main Swimmers-Plot: all Patients with an event irrespective of f/u-time or without an event and f/u-time of >12 months. - Inset: Patients without an event and f/u-time <12 months. - Excluded are 6 patients with only one-time consults (less than 1 month of follow-up time.

**Fig. 3:**
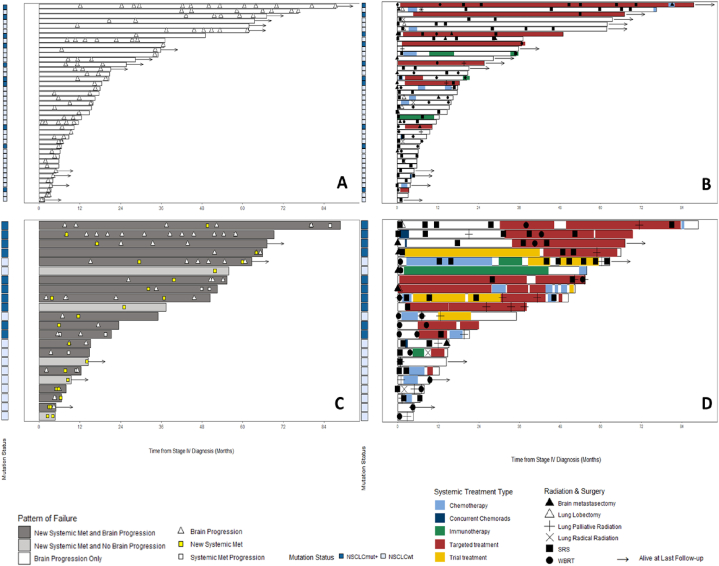
Visualisation of events and associated treatments in swimmers plots. A) Brain-progression events in patients with brain only progression. B) Treatments given in patients with brain-only progression initially and at progression. C) Brain and systemic progression events in patients with at least one systemic event. D) Treatments given in patients with at least one systemic progression initially and at progression.

A pattern of failure analysis revealed a brain-predominant picture for patients with initial BOM (Fig. 2). Forty-one patients (38 %) failed exclusively and usually multiple times in the brain; these patients were primarily treated with repeated local brain interventions. Overall, excluding 1-time consult patients, among those with brain progression, median time-to-first-brain-progression was 7.4 months (NSCLCwt 5.7 months vs 12.1 months in NSCLCmut+; Fig. 3A and B). Twenty-two patients (20 %) developed extracranial metastases; the majority of whom also developed further brain progression. In these patients, median time-to-first-occurrence of systemic metastases was 8.7 months and differed significantly between patients with NSCLCwt versus NSCLCmut + disease (8.5 vs 21 months; Fig. 3C and D; Supplementary Fig. 2).

Patients with initial BOM who later developed extracranial disease were more likely to be younger or have NSCLCmut+ (Supplementary Table 2).Twenty-nine patients died without radiological progression (brain and/or systemic) after initial diagnosis of stage IV disease. Clinical progression and resultant death due to NSCLC was observed in 16, while 6 died of non-cancer-related causes, including postoperative complications or infections. In the 7 remaining patients, the cause of death was unknown.

Overall, 17 patients remained alive and without disease progression at last follow-up; of these, 5 had a <12 months follow-up and 6 were 1-time consults only.

Within all patients who had documented progression, 92 % (58/63) progressed in the brain. Within the entire cohort, 53 % (58/109) progressed in the brain at least once: (48 % (39/82) of NSCLCwt patients and 70 % (19/27) of NSCLCmut + patients).

**Management over the course of disease:** Most patients (86 %) received at least one course of local BrM treatment. Many received multiple courses of SRS or SRS followed by WBRT; the majority received 1–3 courses of SRS (n = 84; 77 %); some received up to 8. Patients with NSCLCmut+ were more likely to receive three or more courses of SRS compared to patients with NSCLCwt (37 % vs 8.5 %, p < 0.01). Seven patients received 2 WBRT courses (Fig. 3B and D).

Overall, 54 patients (50 %) were treated with at least 1 line of systemic therapy; 33 patients (30 %) were never treated systemically; 22 (20 %) patients were referred from outside our institution where data were unavailable) (Fig. 3B and D).

### 3.3 Survival outcomes

**Fig. 4:**
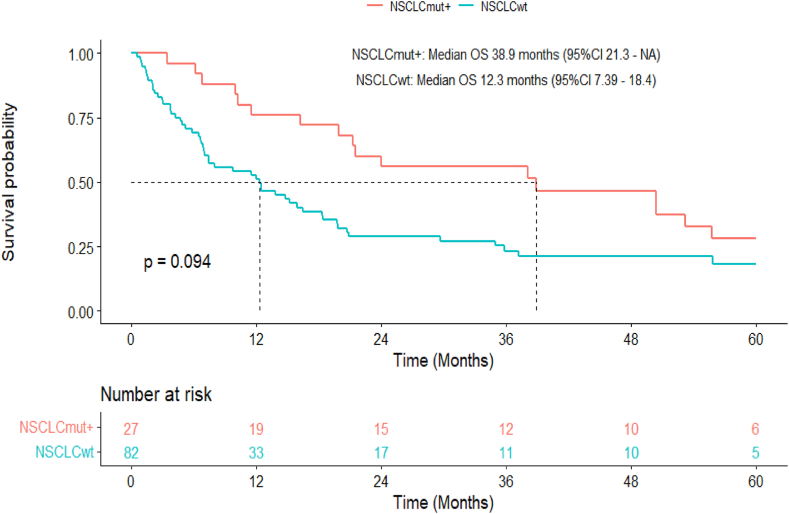
Median Overall Survival in patients with brain-only metastatic disease depending on histology.

With a median follow-up of 14.3 months, median overall survival (OS) in patients with BOM disease was 15.9 months (range 11.5–21.3): 12.3 months (range 7.4–18.4) for NSCLCwt (adenocarcinoma: 12.5 months, other histologies: 12.0) and 38.9 months (range 21.3-NR) for NSCLCmut+ (p = 0.09) (Fig. 4).

**Fig. 5:**
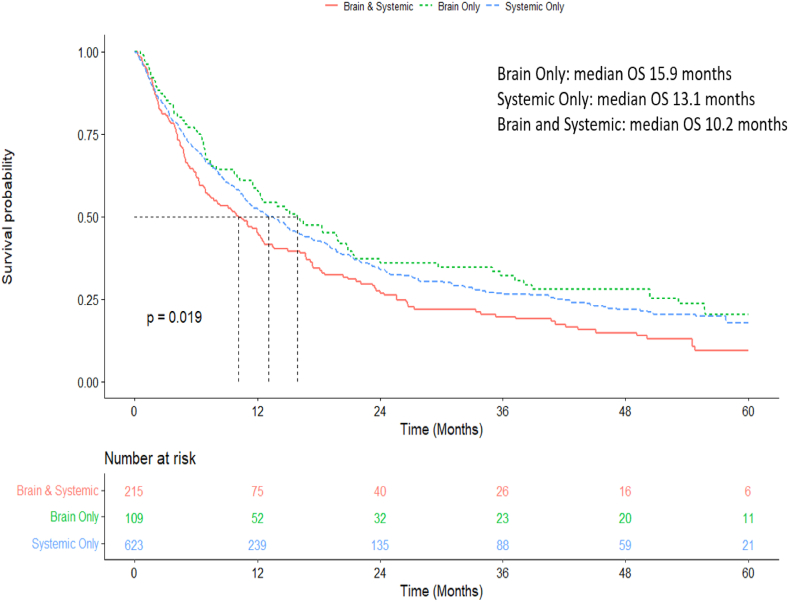
Overall survival from diagnosis of stage IV disease depending on initial metastatic pattern.

Median OS was significantly longer in patients with BOM compared to patients with brain and systemic metastases or systemic only metastases at baseline (15.9 months range 11.5–21.3 vs. 10.2 months, range 7.1-12-7 vs. 13.1 months, range 11.1–15.8) (p = 0.02) (Fig. 5). Median OS was 10.3 months for patients with BOM after initial curative intent treatment,which was borderline-significantly shorter than patients with *de novo* BOM stage IV disease (median OS of 18.4 months; p = 0.07)(Supplementary Fig. 3).

There were no statistically significant differences in OS according to primary tumor treatment univariately (median OS 29.7 vs 16.2 months, p = 0.12) (Supplementary Fig. 4). In contrast to patients with NSCLCwt (median OS 16.5 vs 11.5 months, p = 0.27) (Supplementary Fig. 5A), however, an association, acknowledging for the bias of treatment by indication and the very small sample size, was detected for patients with NSCLCmut+ (median OS 24 months *versus* not reached in months, p = 0.05) (Supplementary Fig. 5B). In multivariable analysis adjusting for age, mutational status, and whether palliative systemic treatment was initiated within 4 months of diagnosis, there was a significant association between survival and primary local treatment in the overall cohort cohort(aHR 0.48 (95%CI 0.26–0.90, p = 0.02)). In a sensitivity analysis, we additionally controlled for T and N stage within patients for whom data was available (n = 72). Treatment to the primary tumor remained significantly associated with OS (HR 0.43 (95 % CI 0.22–0.83, p = 0.012). In a second sensitivity analysis including only patients where, in addition to local treatment to the primary, all BrM were treated locally (n = 70), results were comparable (data not shown).

Twenty-nine percent (n = 32), 22 % (n = 24) and 10 % (n = 11) of patients had a median OS of 2, 3 and 5 years respectively. Median OS > 5 years was observed in both patients with NSCLCmut+ (n = 6) and NSCLCwt (all adenocarcinoma, n = 5). Survival >5 years correlated with age (60.6 vs 67.5 years, p = 0.05), non-Caucasian ethnicity (38 % vs 72 %, p = 0.10), and never smokers (55 vs 20 %, p = 0.02). Number of brain lesions at baseline did not have a significant impact.

## 4 Discussion

In this analysis, we report real-world- outcomes of patients with BOM disease at the time of stage IV diagnosis (either at presentation or as a recurrence). Overall, we observed favourable survival outcomes with a median OS of 15.9 months and 10 % 5-year OS. Initial treatment after diagnosis included local treatment for BrM in most patients, whereas only 39 % of patients received systemic treatment within 4 months of diagnosis; as may be expected, in patients with NSCLCmut+, this percentage was higher (67 %). Interestingly, the majority of patients maintained a brain-predominant metastatic pattern during their disease course; 38 % had brain-progression exclusively while 20 % experienced extracranial and intracranial progression. Whereas only 15 % of patients with NSCLCwt had subsequent extracranial failure t), 37 % of patients in the BOM NSCLCmut + cohort developed extracranial metastases over the course of their disease.

In a multi-institutional retrospective database analysis led by Sperduto in 2017median OS for NSCLC with brain metastases with and without extracranial metastases was 12 months and differed significantly depending on molGPA score [5]. Among our cohort of BOM NSCLC, median OS was 15.7 month. The recently updated lung-specific GPA included PD-L1 expression, which was not systematically assessed in our cohort because the majority of our patients were treated before widespread use of checkpoint inhibitors [6]. Nevertheless, our data suggests a favourable prognosis for patients with BOM.

In our analysis, we demonstrated a persistent brain-predominant metastatic pattern requiring repetitive local treatments in patients with BOM at baseline. The majority of patients were managed with SRS. Some experienced prolonged survival, over which they received several courses of radiotherapy, primarily SRS. Although guidelines recommend SRS for up to 4 BrM and to consider in >4 BrM, when a CNS-penetrating systemic therapy is available [7], the cut-off of 4 was challenged by Yamamoto et al., who analyzed outcomes among patients treated with SRS alone for up to 10 lesions. They did not detect a difference in OS among patients with 2–4 lesions versus 5–10 [11]. The brain-predominant failure pattern observed in our cohort emphasizes the need for regular brain imaging after initial treatment and supports the use of cognition-sparing modalities such as SRS initially to reduce the potential negative effects of WBRT. Intrestingly in a recently published analysis in patients with ALK and EGFR positive NSCLC with brain metastases and treatment with newer generation TKIs, upfront SRS to brain metastases was associated with imptroved time-to-CNS progression and local CNS control, however no benefit with regards to OS was observed. On the other hand, for patients with NSCLCwt treated with less CNS-penetrating systemic treatment options than are available for NSCLCmut+, in view of this brain-predominant failure pattern, systemic therapy may be postponed in some patients in favour of repetitive local treatments. Only 15 % of patients with NSCLCwt and BOM developed subsequent extracranial metastases compared to 37 % of patients with NSCLCmut+. This difference in failure pattern could be partly driven by improved survival among NSCLCmut+, future research may consider evaluating the risk of developing brain metastasis with death as a competing risk event. That said, within those with at least 1 progression, median time to developing an extracranial metastasis in patients with NSCLCwt was 8.5 months compared to 21 months in patients with NSCLCmut+. Therefore, there also remains the possibility that underlying biological differences wrought by the presence or absence of driver mutations lead to distinct metastatic patterns and suggests that different imaging monitoring strategies might be applied based on EGFR-mutation/ALK-rearrangement status.

There is a growing body of evidence mainly from phase II and retrospective studies suggesting a benefit for radical treatment to all metastatic sites in addition to the primary tumor in patients with oligometastatic NSCLC [10]. Some studies support this approach in NSCLCwt [12,13] whereas others suggest a benefit in patients with EGFR-mutations [14,15]. However, randomized phase III data are lacking and results of currently recruiting trials such as NRG-LU002 are pending. In our selected population, while we did not detect a significant association between primary tumor treatment and overall survival in the NSCLCwt population in a univariate model, we did observe statistically greater survival in patients with NSCLCmut + who had their primary treated locally (in the majority of cases in conjunction with local treatment of brain metastases). However, when assessing the association between local tumor treatment and overall survival in the multivariate model, we did observe a significant association within the overall cohort. Nevertheless, interpretation of these results should be taken with caution due to the relatively small sample size, the retrospective nature of this analysis, and a lack of information on whether local therapy was given due to clinical symptoms or other reasons. Furthermore, the majority of our patients in the NSCLCwt cohort were treated before widespread use of checkpoint inhibitors and therefore it is unclear to what extent our results can be extrapolated to the current landscape.

Our analysis has several limitations, including its retrospective design and data collection. Potential selection bias may exist as this study was conducted at a single academic institution. Radiographic responses were assessed retrospectively by local investigators and no formal tumor assessment by RECIST was performed. We were not able to obtain tumor size and resection margins in all patients due to a number of patients initially diagnosed outside our institution, and treatment decisions were not randomized, thus we acknowledge that some potential confounders may not be controlled for and there presents potential baseline risk differences between groups in our evaluation of the effect of primary tumor treatment. Due to the small sample size of this relatively rare population, not all relevant confounders could be included in the main model, and additional sensitivity analysis models should be interpreted with the acknowledgement of small sample size issues. Future studies can look to further investigate the effect of primary tumor treatment within NSCLCwt and NSCLCmut + populations with a larger sample size.

## 5 Conclusion

In summary, our analysis demonstrates favourable survival outcomes in patients with Stage IV NSCLC and BOM. We observed significant differences in metastatic failure patterns according to mutational status. More patients with NSCLCmut + developed subsequent extracranial metastases compared to patients with NSCLCwt. Furthermore, primary tumor treatment was associated with improved survival in patients with NSCLCmut+.

## Ethics statement

• This study was reviewed and approved by University Health Network Research Ethics Board with the approval number 19–5099.12; date of approval: October 2019
• Informed consent was not required for this study because of the retrospective data collection.

## Funding

This research did not receive any specific grant from funding agencies in the public, commercial, or not-for-profit sectors.

## Data availability statement

Has data associated with your study been deposited into a publicly available repository?: No, all relevant data is shown in the manuscript and/or supplementary material.

## CRediT authorship contribution statement

**Sabine Schmid:** Writing – original draft, Project administration, Methodology, Investigation, Data curation, Conceptualization. **Miguel Garcia:** Writing – review & editing, Investigation, Data curation. **Luna Zhan:** Writing – review & editing, Formal analysis, Data curation. **Sierra Cheng:** Writing – review & editing, Formal analysis, Data curation. **Khaleeq Khan:** Investigation, Data curation. **Maisha Chowdhury:** Investigation, Data curation. **Amir Sabouhanian:** Investigation, Data curation. **Joshua Herman:** Investigation, Data curation. **Preet Walia:** Investigation, Data curation. **Evan Strom:** Investigation, Data curation. **M. Catherine Brown:** Writing – review & editing, Methodology, Data curation. **Devalben Patel:** Project administration, Investigation, Data curation. **Wei Xu:** Writing – review & editing. **Frances A. Shepherd:** Writing – review & editing. **Adrian G. Sacher:** Writing – review & editing. **Natasha B. Leighl:** Writing – review & editing. **Penelope A. Bradbury:** Writing – review & editing. **Geoffrey Liu:** Writing – review & editing, Supervision, Conceptualization. **David Shultz:** Writing – review & editing, Supervision, Conceptualization.

## Declaration of competing interest

The authors declare the following financial interests/personal relationships which may be considered as potential competing interests:Sabine Schmid reports a relationship with MSD, Merck, BMS, AstraZeneca (all paid to institution) that includes: consulting or advisory. Sabine Schmid reports a relationship with 10.13039/100004325AstraZeneca, 10.13039/100014554Janssen, 10.13039/100008021BMS, 10.13039/100014554Janssen (all paid to institution) that includes: funding grants. Sabine Schmid reports a relationship with Swiss Cancer League that includes: funding grants. Sabine Schmid reports a relationship with Takeda, MSD, Amgen that includes: travel reimbursement. Geoffrey Liu reports a relationship with Boehringer Ingelheim, 10.13039/100004325AstraZeneca, 10.13039/100008021Bristol Myers Squibb, 10.13039/100004334Merck, 10.13039/100016040Takeda, 10.13039/100004319Pfizer, Hoffman La Roche, 10.13039/100006483Abbvie, 10.13039/100004755EMD Serono, Eli Lilly (all insitutional) that includes: consulting or advisory and funding grants. If there are other authors, they declare that they have no known competing financial interests or personal relationships that could have appeared to influence the work reported in this paper.
