# Supplementary material for: Outcomes with non-small cell lung cancer and brain-only metastasis
Source: Heliyon. 2024 Aug 29;10(17):e37082. doi: 10.1016/j.heliyon.2024.e37082 (PMC11408029; doi:10.1016/j.heliyon.2024.e37082)
Supplement: Multimedia component 1 [file mmc1.docx]

**Data supplement:**

**Supplementary Figures:**

**Supplementary Figure 1: Treatment patterns at initial diagnosis (within 4 months of stage IV diagnosis) within 101 patients receiving at least 1 treatment depending on mutational status**

1. Wildtype (NSCLCwt) population
2. EGFR-mutated/ALK-rearranged (NSCLCmut+) population

**
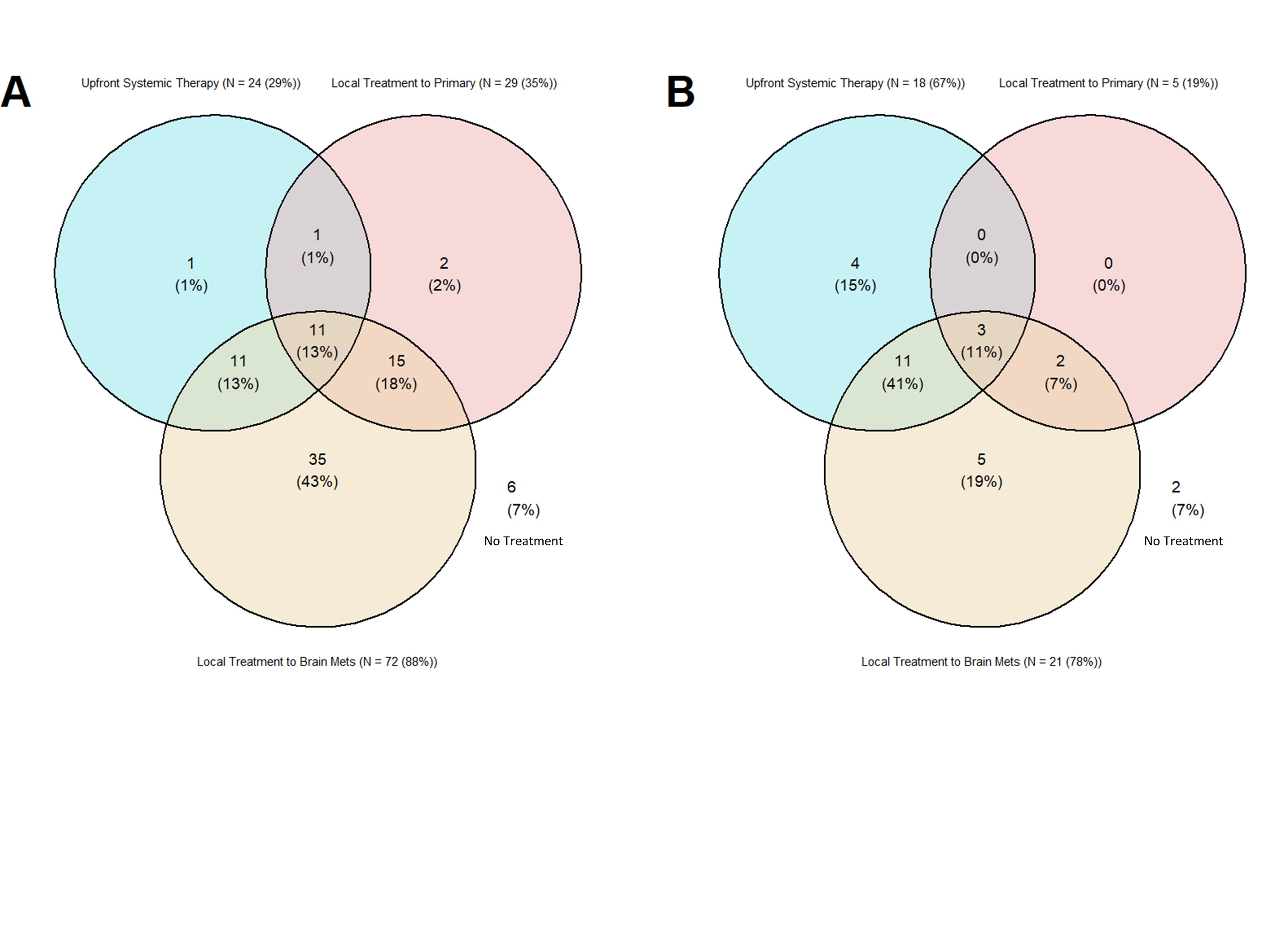
**

**Supplementary Figure 2: Type of metastatic progression depending on histology**


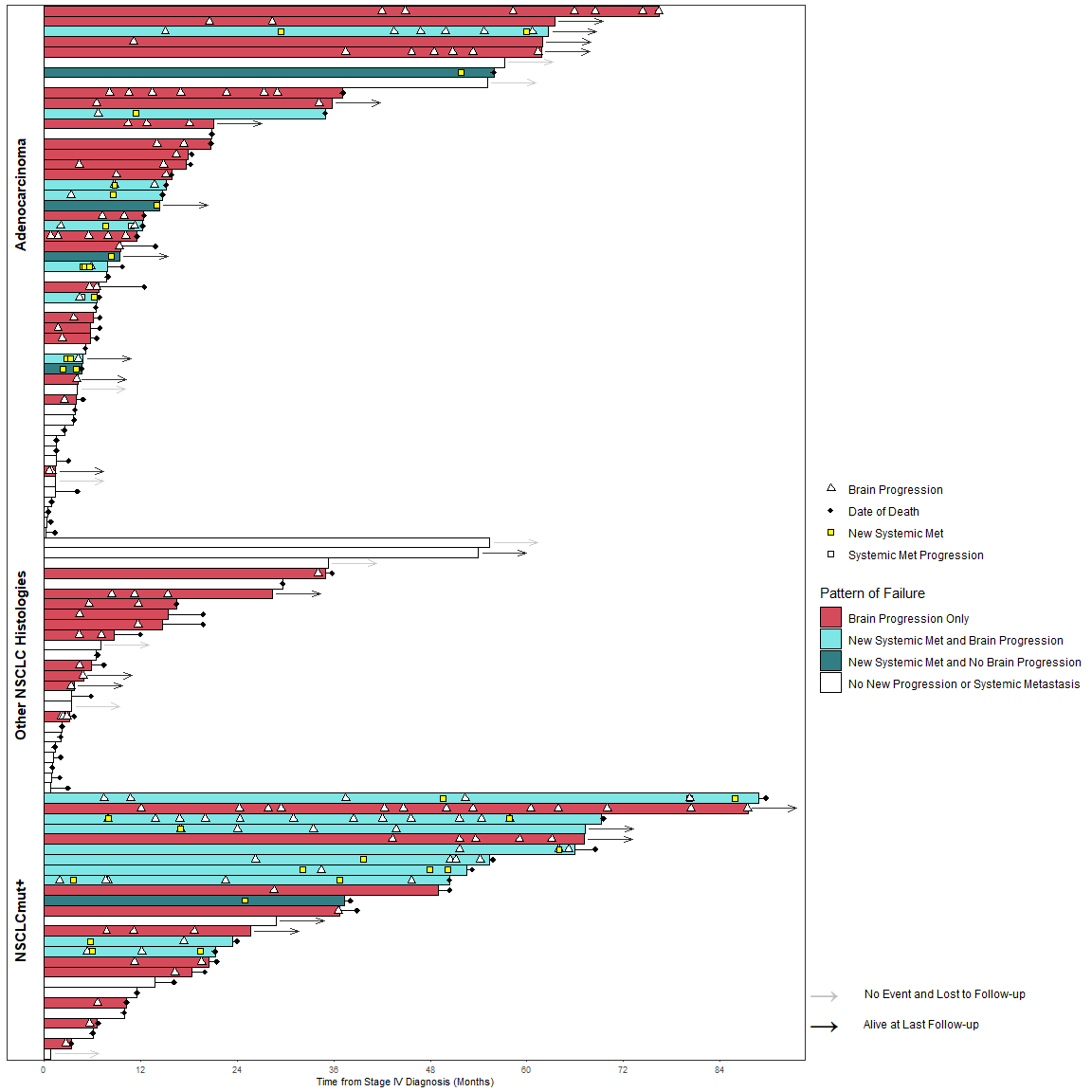


**Supplementary Figure 3: Median Overall Survival in patients with brain-only metastatic disease depending on whether they presented with *de novo* stage IV disease or stage IV disease after initial curative intent treatment (metastatic recurrence)**


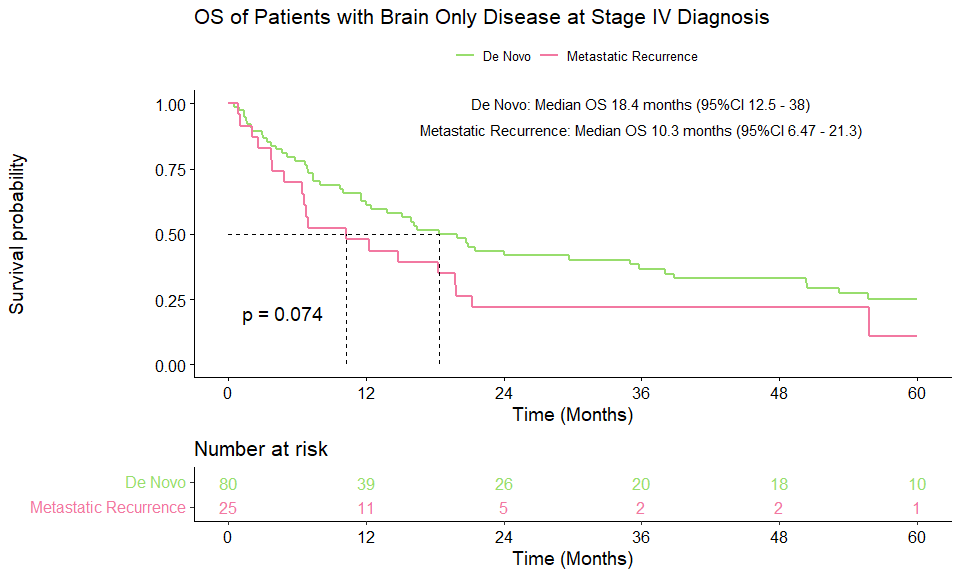


**Supplementary Figure 4: Median Overall survival in patients with de novo brain-only disease (n=80) depending on whether their primary tumor was treated (blue line) or not treated (red line)**


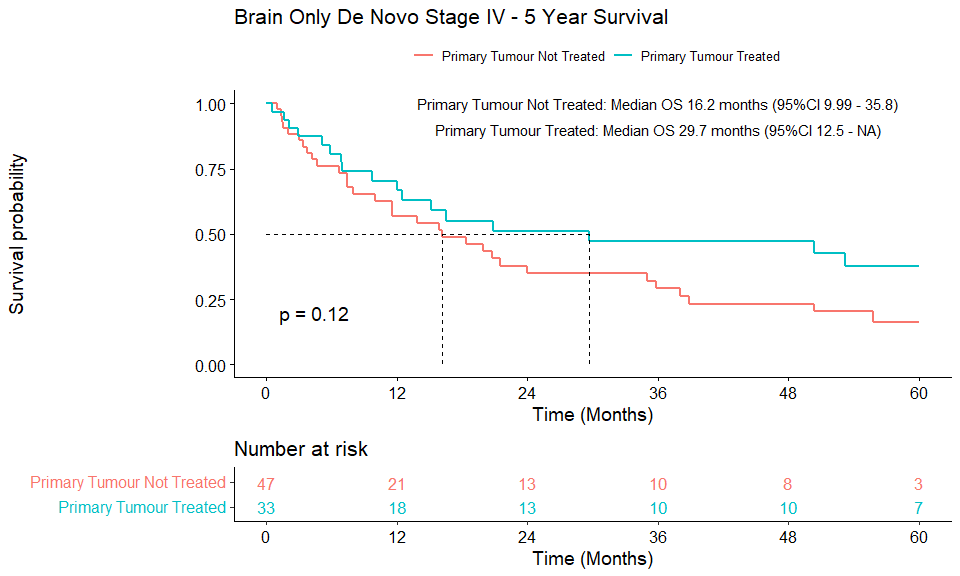


**Supplementary Figure 5: Median Overall survival in patients with de novo brain-only disease depending on whether their primary tumor was treated (blue line) or not treated (red line)**

A: NSCLCwt

B: NSCLCmut+

**
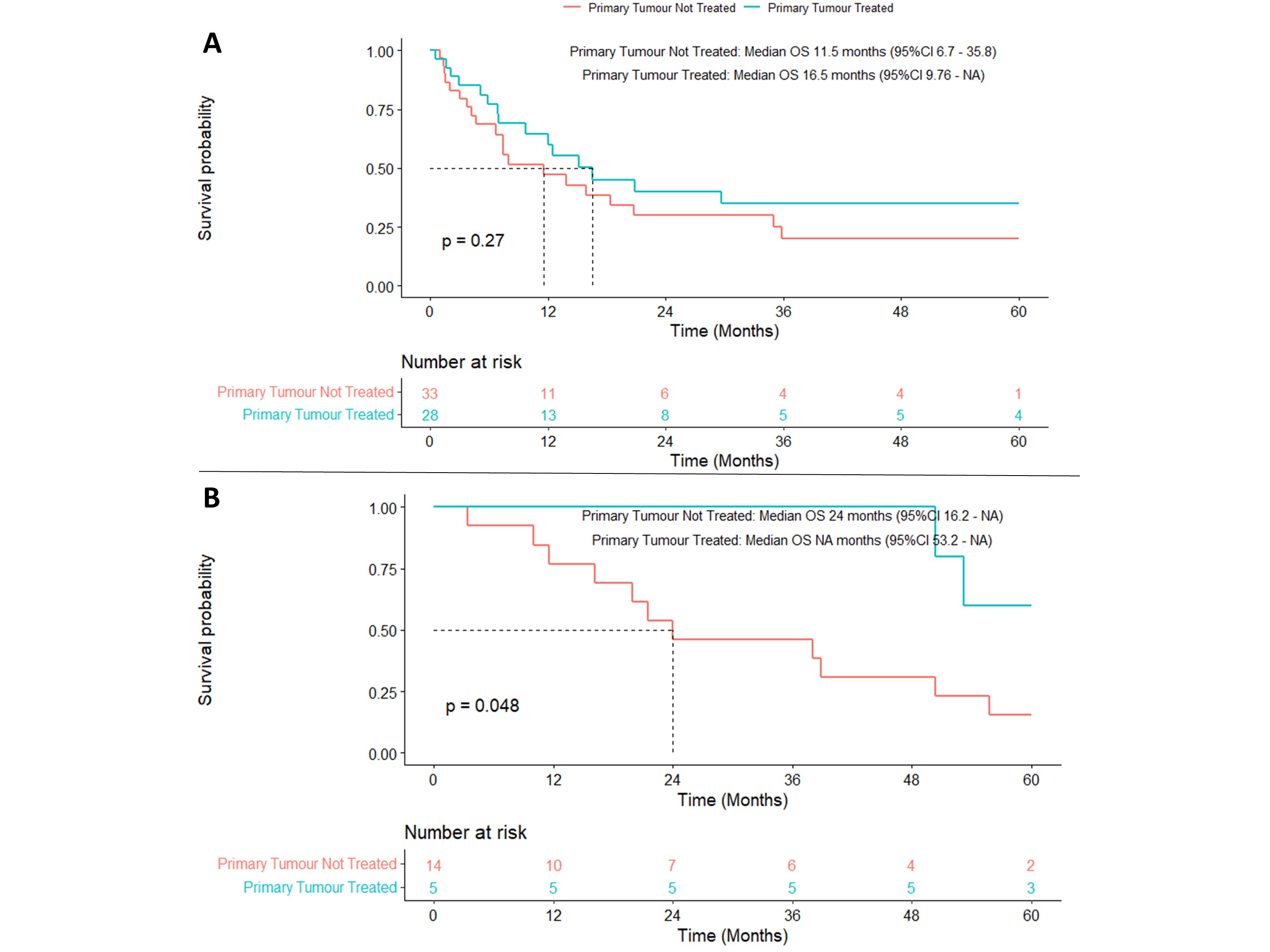
**

**Supplementary Tables:**

**Supplementary Table 1: Baseline characteristics of patients with de novo brain metastatic disease (n=80) depending on whether their primary tumor was treated locally or not**

| Covariate | Category | Overall cohort | Primary not treated | Primary treated | p-value |
| --- | --- | --- | --- | --- | --- |
| Total N (%) |  | 80  (100) | 47  (59) | 33  (41) |  |
| Age at stage IV | Median years (IQR) | 66.2  (59.4-72.8) | 65.2  (59.8-72.6) | 69.4  (59.5-73.1) | 0.5 |
| Sex | Female  Male | 42 (53)  38 (48) | 23 (49)  24 (51) | 19 (58)  14 (42) | 0.5 |
| Ethnicity | Asian  Caucasian  Other  Missing | 14 (26)  37 (69)  3 (6)  26 | 10 (32)  19 (61)  2 (7)  16 | 4 (17)  18 (78)  1 (4)  10 | 0.5 |
| Smoking Status | Ever  Never  Missing | 62 (82)  14 (18)  4 | 36 (84)  7 (16)  4 | 26 (79)  7 (21)  0 | 0.8 |
| ECOG | 0  1  2  3  Missing | 13 (25)  21 (40)  11 (21)  7 (14)  28 | 8 (29)  11 (39)  3 (11)  6 (21)  19 | 5 (21)  10 (42)  8 (33)  1 (4)  9 | 0.1 |
| Histology | Adenocarcinoma wildtype  EGFR mutated/ALK rearranged  Squamous Cell  Large Cell carcinoma | 41 (51)  19 (24)  15(19)  5 (6) | 22 (47)  14 (30)  8 (17)  3 (6) | 19 (58)  5 (15)  7 (21)  2 (6) | 0.5 |
| Number of brain lesions | 1-2  3-9  >/=10 | 46 (58)  19 (24)  15 (19) | 22 (47)  13 (28)  12 (26) | 24 (73)  6 (18)  3 (9) | **0.05** |
| Initial local brain treatment | Yes  No | 70 (88)  10 (13) | 40 (85)  7 (15) | 30 (91)  3 (9) | 0.51 |
| Systemic treatment within 4 months of diagnosis | Yes  No  Missing | 34 (46)  40 (54)  6 | 19 (45)  23 (55)  5 | 15 (47)  17 (53)  1 | 1.0 |
| T Stage | 0-1  2  3-4  Missing | 19 (26)  29 (40)  25 (34)  7 | 13 (31)  18 (43)  11 (26)  5 | 6 (19)  11 (35)  14 (45)  2 | 0.24 |
| T Stage | Median (IQR) | 2.0 (1.0 to 3.0) | 2.0 (1.0 to 2.8) | 2.0 (2.0 to 3.0) | 0.08 |
| N Stage | 0  1  2  3  Missing | 26 (35)  5 (7)  34 (45)  10 (13)  5 | 15 (36)  4 (10)  16 (38)  7 (17)  5 | 11 (33)  1 (3)  18 (55)  3 (9)  0 | 0.42 |
| N stage | Median (IQR) | 2.0 (0.0 to 2.0 | 2.0 (0.0 to 2.0 | 2.0 (0.0 to 2.0 | 0.93 |

**Supplementary Table 2: Baseline characteristics of patients with brain-only failure compared to patients developing extracranial metastases in the course of the disease**

- Only patients with at least one event (brain and/or systemic PD) are included in this table.
- 46 patients without event at last f/u or before death are excluded (including patients with only clinical PD without imaging before death)
  - 29 patients with death without event: death to NSCLC/clinical PD in 16 patients, death due to other causes in 6 patients, death due to unknown cause in 7 patients
  - 2 patients without an event and alive at last f/u
  - 15 patients without event and lost to f/u

| Covariate | Category | Overall cohort (all patients with event) | Brain only PD | Systemic (+/- brain) PD | p-value |
| --- | --- | --- | --- | --- | --- |
| Total N (%) |  | 63 | 41 | 22 |  |
| Age (stage IV) | Median years (IQR) | 63.5  (56.2-70.2) | 67.3  (57.9-71.6) | 59.8  (53.1-64.9) | **0.03** |
| Sex | Female  Male | 32 (51)  31 (49) | 18 (44)  23 (56) | 14 (64)  8 (36) | 0.22 |
| Ethnicity | Asian  Caucasian  Other  Missing | 12 (26)  30 (64)  5 (11)  16 | 7 (24)  19 (66)  3 (10)  12 | 5 (28)  11 (61)  2 (11)  4 | 0.95 |
| ECOG | 0  1  2  3  Missing | 13 (28)  23 (49)  8 (17)  3 (6)  16 | 8 (28)  14 (48)  6 (21)  1 (3)  12 | 5 (28)  9 (50)  2 (11)  2 (11)  4 | 0.65 |
| Smoking Status | Ever  Never  Missing | 46 (74)  16 (26)  1 | 31 (78)  9 (23)  1 | 15 (68)  7 (32)  0 | 0.62 |
| Histology | Adenocarcinoma wt  EGFR/ALK pos.  Squamous Cell  Large Cell | 33 (52)  20 (32)  5 (8)  5 (8) | 21 (51)  10 (24)  5 (12)  5 (12) | 12 (55)  10 (46)  0 (0)  0 (0) | **0.06** |
| Stage at initial diagnosis | I  II  III  IV | 1 (2)  5 (8)  11 (18)  46 (73) | 1 (2)  5 (12)  7 (17)  28 (68) | 0 (0)  0 (0)  4 (18)  18 (82) | 0.31 |
| Number of brain lesions at diagnosis | 1-2  3-9  >10  missing | 36 (58)  17 (27)  9 (15)  1 | 26 (65)  8 (20)  6 (15)  1 | 10 (46)  9 (41)  3 (14)  0 | 1.00 |
| Initial local brain treatment | Yes  No | 53 (82)  10 (18) | 34 (83)  7 (17) | 19 (86)  3 (14) | 1.00 |
| Initial local treatment to primary | Yes  No | 21 (33)  42 (67) | 11 (27)  30 (73) | 10 (45)  12 (55) | 0.14 |
| Systemic treatment within 4 months of diagnosis | Yes  No  Missing | 29 ()  32 (53)  2 | 16 (41)  23 (59)  2 | 13 (59)  9 (41)  0 | 0.28 |
